# Benchmarking of computational methods for m6A profiling with Nanopore direct RNA sequencing

**DOI:** 10.1093/bib/bbae001

**Published:** 2024-01-26

**Authors:** Simone Maestri, Mattia Furlan, Logan Mulroney, Lucia Coscujuela Tarrero, Camilla Ugolini, Fabio Dalla Pozza, Tommaso Leonardi, Ewan Birney, Francesco Nicassio, Mattia Pelizzola

**Affiliations:** Center for Genomic Science of IIT@SEMM, Fondazione Istituto Italiano di Tecnologia (IIT), Milan, Italy; Center for Genomic Science of IIT@SEMM, Fondazione Istituto Italiano di Tecnologia (IIT), Milan, Italy; Center for Genomic Science of IIT@SEMM, Fondazione Istituto Italiano di Tecnologia (IIT), Milan, Italy; European Molecular Biology Laboratory, European Bioinformatics Institute, Hinxton, Cambridgeshire, U.K; Epigenetics and Neurobiology Unit, European Molecular Biology Laboratory (EMBL), Rome, Italy; Center for Genomic Science of IIT@SEMM, Fondazione Istituto Italiano di Tecnologia (IIT), Milan, Italy; Center for Genomic Science of IIT@SEMM, Fondazione Istituto Italiano di Tecnologia (IIT), Milan, Italy; Center for Genomic Science of IIT@SEMM, Fondazione Istituto Italiano di Tecnologia (IIT), Milan, Italy; Center for Genomic Science of IIT@SEMM, Fondazione Istituto Italiano di Tecnologia (IIT), Milan, Italy; European Molecular Biology Laboratory, European Bioinformatics Institute, Hinxton, Cambridgeshire, U.K; Center for Genomic Science of IIT@SEMM, Fondazione Istituto Italiano di Tecnologia (IIT), Milan, Italy; Center for Genomic Science of IIT@SEMM, Fondazione Istituto Italiano di Tecnologia (IIT), Milan, Italy; Department of Biotechnology and Biosciences, University of Milano-Bicocca, Milan, Italy

**Keywords:** RNA modifications, N6-methyladenosine, Nanopore, dRNA-seq, benchmarking, machine learning

## Abstract

N6-methyladenosine (m6A) is the most abundant internal eukaryotic mRNA modification, and is involved in the regulation of various biological processes. Direct Nanopore sequencing of native RNA (dRNA-seq) emerged as a leading approach for its identification. Several software were published for m6A detection and there is a strong need for independent studies benchmarking their performance on data from different species, and against various reference datasets. Moreover, a computational workflow is needed to streamline the execution of tools whose installation and execution remains complicated. We developed NanOlympicsMod, a Nextflow pipeline exploiting containerized technology for comparing 14 tools for m6A detection on dRNA-seq data. NanOlympicsMod was tested on dRNA-seq data generated from *in vitro* (un)modified synthetic oligos. The m6A hits returned by each tool were compared to the m6A position known by design of the oligos. In addition, NanOlympicsMod was used on dRNA-seq datasets from wild-type and m6A-depleted yeast, mouse and human, and each tool’s hits were compared to reference m6A sets generated by leading orthogonal methods. The performance of the tools markedly differed across datasets, and methods adopting different approaches showed different preferences in terms of precision and recall. Changing the stringency cut-offs allowed for tuning the precision-recall trade-off towards user preferences. Finally, we determined that precision and recall of tools are markedly influenced by sequencing depth, and that additional sequencing would likely reveal additional m6A sites. Thanks to the possibility of including novel tools, NanOlympicsMod will streamline the benchmarking of m6A detection tools on dRNA-seq data, improving future RNA modification characterization.

## INTRODUCTION

RNA molecules are known to be decorated by more than 160 different chemical modifications, which can be found on both the nitrogenous base and the ribose sugar [1] and have profound consequences on the fate of coding and non-coding RNA species [2]. Many studies have been conducted to investigate the prevalence, transcriptome distribution, and functional role of N6-methyladenosine (m6A), the most abundant internal modification of eukaryotic coding transcripts [3, 4]. m6A is a reversible and dynamic mark deposited by methyltransferases, mainly at the RRACH (R = A/G, H = U/A/C) consensus motif, removed by demethylases, and recognized by specific effector proteins which mediate a large set of effects [3]. Indeed, m6A has been shown to markedly impact RNA metabolism, including processing, degradation, translation and localization [5, 6]. Additionally, m6A has been shown to be involved in wide-ranging roles of gene expression regulation, both in physiological conditions, including cellular differentiation, meiosis, heat stress response, gametogenesis and neurons activity [7], and pathological conditions, such as viral infection and several types of cancer [8, 9].

The development of various methods for quantification and mapping of RNA modifications was pivotal for the surge of RNA modifications research in the last decade [10]. In particular, various approaches based on high-throughput sequencing were developed for m6A profiling that rely on immunoprecipitation of modified molecules (e.g. MeRIP-seq [11], m6A-seq [12], m6A-seq2 [13], miCLIP [14], miCLIP2 [15] and m6A-LAIC-seq [16]), or on biochemical treatments that leave characteristic footprints on the cDNA, depending on the presence of the RNA modification of interest (e.g. PA-Seq [17], MAZTER-seq [18], m6A-REF-seq [19], DART-seq [20], m6A-SEAL-seq [21], m6A-label-seq [22], GLORI [23] and m6A-SAC-seq [24]). However, specific antibodies, enzymes and chemical compounds are currently available only for a limited set of RNA modifications, they can have low specificity, they are typically semi-quantitative, they are inadequate for profiling more than one modification simultaneously [25, 26], and they lack isoform and single molecule-resolution. Recently, Oxford Nanopore Technologies (ONT) launched a platform to directly sequence native RNA molecules (dRNA-seq) [27]. The electric signal recorded by ONT sequencing platform was shown to be altered by the presence of RNA modifications [27–29]. This paved the way to the single-molecule and single-base characterization of RNA modifications and prompted the development of computational tools to profile m6A from dRNA-seq data [30, 31]. These tools can be divided into two main groups: (i) single-condition tools, which require training a machine learning algorithm capable of distinguishing between modified and unmodified nucleotides and (ii) multi-condition tools, which require sequencing an additional condition devoid of the modification(s) of interest. The latter is typically generated through knock-down, knock-out or pharmacological inhibition of methyltransferases or by *in vitro* transcription. Tools can be additionally divided depending on whether they rely on the ionic current signal, or errors in the base calling. Furthermore, some tools can provide the modification stoichiometry, and some can achieve base-level or single-molecule resolution depending on whether they work in the genome or transcriptome reference space [32].

More than a dozen tools were already published, and others are likely to be released in the near future. These tools are often complex to install and have high demands in terms of storage and computing power [33]. Although each software has already been compared against selected tools at the time of publication, only recently a study covered multiple tools for m6A detection on dRNA-seq data focusing on the mouse epitranscriptome [34]. Altogether, users are left with limited guidance on how to prioritize the choice of the tool, on how to set significance cut-offs, and on how to coherently test additional novel tools.

To address these needs and open questions, we developed NanOlympicsMod, a computational workflow designed to maximize reproducibility and portability in the comparative assessment of dRNA-seq m6A detection tools. We used NanOlympicsMod to execute and compare 14 tools on four different dRNA-seq datasets differing in terms of synthetic/biological origin, transcriptome size and coverage depth. The results were benchmarked against reference m6A sets obtained from established orthogonal techniques. This study showed a remarkable heterogeneity in the performances of the tools, underlying the importance of a critical selection of the software and cut-off settings depending on the desired precision and recall targets.

## RESULTS

### The NanOlympicsMod benchmarking framework

**Figure 1:**
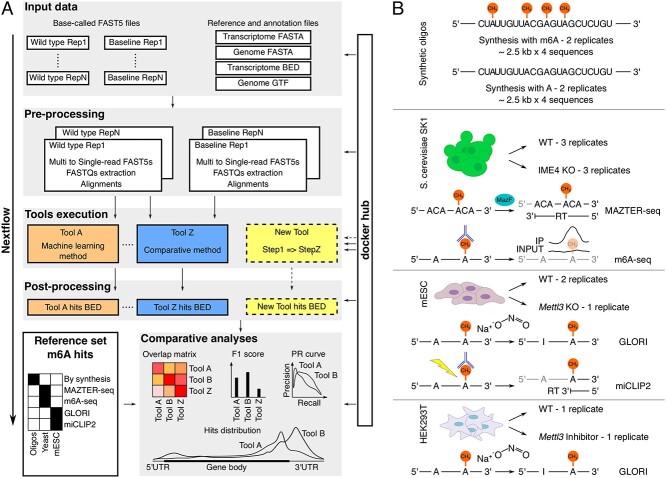
The NanOlympicsMod workflow and adopted datasets. (**A**) Schema of NanOlympicsMod, including input data, pre-processing steps, tools execution, post-processing and comparative analyses. (**B**) Experimental design for the four different datasets analysed by NanOlympicsMod; the methods used to generate the reference set of m6A hits in yeast and mouse are illustrated.

We set up NanOlympicsMod, a framework to benchmark software profiling RNA modifications on Nanopore dRNA-seq data. This framework was adopted for the comparative evaluation of 14 computational tools (Supplementary Table 1, see Supplementary Data available online at http://bib.oxfordjournals.org/) and was designed to facilitate the inclusion and test of additional tools. NanOlympicsMod includes a computational pipeline based on Nextflow [35] workflow manager, and was applied to four different dRNA-seq datasets, together with a set of corresponding reference sets of m6A hits generated by various short-reads sequencing based techniques (Figure 1).

NanOlympicsMod relies on Nextflow, guaranteeing portability across platforms and support for different job schedulers, and adopts Docker and Singularity container technologies, removing the need to install required software dependencies and ensuring reproducibility. The pipeline includes a pre-processing module, preparing the input files required by each tool, a module for the parallel execution of the tools, a post-processing module converting the output of the tools in a common format, and a module implementing a set of analyses for the assessment of the tools results, their mutual concordance, and their agreement with independent reference sets of m6A hits (Figure 1A).

We applied NanOlympicsMod to three recently released dRNA-seq datasets for the profiling of m6A on synthetic RNAs, yeast, and mouse transcriptomes, and to a dRNA-seq dataset produced in the context of this work for the profiling of m6A on human transcriptome (Figure 1B and Supplementary Table 2, see Supplementary Data available online at http://bib.oxfordjournals.org/). Each dataset includes an m6A-depleted condition to be used for the comparative tools. The datasets are fully described in the Supplementary data.

Various matching reference sets of m6A hits were considered that encompass different methods for the profiling of the mark, either relying or not on m6A-specific antibodies, including m6A-seq and MAZTER-seq for yeast, miCLIP2 and GLORI for mouse and GLORI for human (Figure 1B and Supplementary Table 3, see Supplementary Data available online at http://bib.oxfordjournals.org/).

### Computational requirements, number and location of hits

We ran all the 14 considered tools (Supplementary Table 1, see Supplementary Data available online at http://bib.oxfordjournals.org/) on the four test datasets (Figure 1). For some of the tools we were not able to complete the analysis or to obtain exploitable results in some of the datasets. Eventually, we were able to complete the analysis for 12 tools in the synthetic oligos dataset, 9 tools in the yeast dataset, 11 tools in the mouse dataset and 11 tools in the human dataset. For nine of these tools, we were able to obtain results in all four datasets. We were unable to complete the analysis in any of the four datasets with one tool, nanoDoc. Each software had different computational requirements, which were influenced by the size of the reference and/or the amount of available sequencing reads (Supplementary Figure S1, see Supplementary Data available online at http://bib.oxfordjournals.org/).

**Figure 2:**
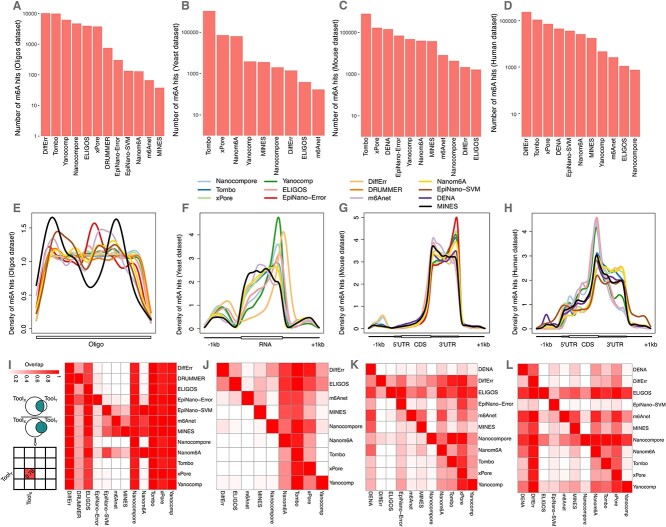
Key results executing the tools with default settings. (**A**) Number of hits detected by NanOlympicsMod for each tool in the Oligos dataset. (**B**) As (**A**) for the yeast dataset. (**C**) As (**A**) for the mouse dataset. (**D**) As (**A**) for the human dataset. (**E**) Distribution of m6A hits for each tool along the synthetic oligos. (**F**) as (**E**) for the yeast metagene. (**G**) as (**E**) for the mouse metagene. (**H**) as (**E**) for the human metagene. (**I**) Heatmap reporting the overlap of m6A hits for each pair of tools executed with default settings on the oligos dataset. The value in a cell represents, for each pair of tools, the proportion of hits in common to the number of hits of the tool on the row (see the schema on the left of the panel). (**J**) As in (**I**) for the yeast dataset. (**K**) As in (**I**) for the mouse dataset. (**L**) As in (**I**) for the human dataset.

The various tools differ in terms of parameters that can be tuned to set significance cut-offs or filtering thresholds, thus defining the stringency of the analysis, and each tool has its own default value for these settings (Supplementary Table 4, see Supplementary Data available online at http://bib.oxfordjournals.org/). We initially decided to run each tool with their respective default settings. In these conditions the tools returned a number of hits that varied by several orders of magnitude, ranging from less than one hundred to more than 1e5 hits. The tools ranking in terms of numerosity of the hits was relatively consistent across the yeast, mouse and human datasets (Figure 2A-D).

The patterning of m6A hits returned by each tool was determined along the length of the synthetic oligos, and along a meta-gene for the yeast, mouse and human datasets. For the modified oligos, all adenosines were replaced by m6A resulting in an even distribution of the modification across the entire RNA length. Given that the sequencing coverage for this dataset is rather uniform, we expected the m6A hits returned by the tools to be uniformly distributed. Indeed, most of the tools returned a relatively flat profile of m6A hits along the oligos. Only MINES, m6Anet, EpiNano-SVM and EpiNano-Error favoured specific parts of the oligos (Figure 2E). In yeast, mouse and human, the m6A hits returned by most of the tools were enriched at 3′ ends and at the stop codon, in agreement with the known location of m6A marks for these species [4]. Only MINES and m6Anet found the m6A hits for the yeast dataset enriched in the mid part of the coding region (Figure 2F-H).

### Overlap among the tools

Once we determined the number and location of m6A hits for each tool, we compared them across the tools. Since Nanopore-based analyses are reflecting the combined influence of a k-mer of bases, typically a 5-mer, it is not trivial to assign m6A hits to specific bases, especially when k-mers contain multiple As. For this reason, we decided to bin the query space (i.e. the oligos or the transcribed portion of yeast, mouse and human genomes). This also allowed to avoid penalizing the concordance between tools whose m6A hits might be separated by a few bases and to avoid inflating the concordance between tools which report multiple adjacent hits for a single modification event. We then determined, for each pair of tools, the overlap of bins with at least one identified m6A. In the oligos dataset, the tools that identified the most m6A sites (> 1000) were in very good agreement among each other, with an average overlap of 0.93 (Figure 2I). The same was observed for the tools with fewer identified m6A sites, in the order of dozens, whose sites were largely a subset of the tools with a higher number of sites. Similar results were obtained for yeast, mouse and human (Figure 2J-L), where the m6A sites identified by the tools with fewer calls were largely confirmed by the sites of the tools with the largest number of calls. The tools that completed the analysis on both yeast and mouse datasets had similar mutual concordance.

### Agreement with reference sets of m6A sites

Once we established that the pattern of m6A hits was plausible and assessed that the tools concordance was largely driven by the number of identified sites, we set to evaluate the tools precision and recall. To this end, the various datasets are differently informative. The distribution of m6A marks in yeast, mouse and human is dictated by the *in vivo* constraints, yet there is no ground truth about the location of the marks. On the contrary, the distribution of m6A marks in the synthetic oligo dataset is artificial, yet their location is known by design.

**Figure 3:**
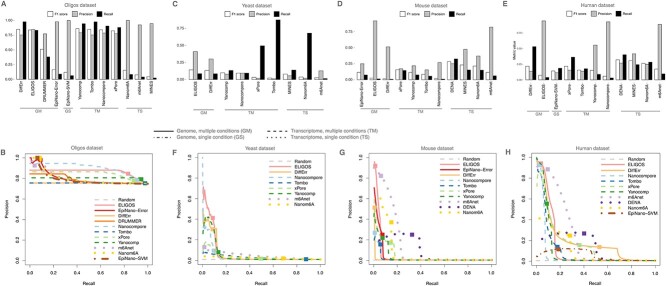
Agreement with reference sets of m6A hits. (**A**) Precision, recall and F1 score for each tool executed at default conditions on the oligos dataset. According to Supplementary Table 1, GM and TM identify tools working on the genome (G) or transcriptome (T) space and require multiple conditions, respectively. GS and TS identify tools working on the genome (G) or transcriptome (T) space and requiring a single condition, respectively. (**B**) Precision and recall curves at different cut-off values for the tools indicated in (A) on the oligos dataset; for each tool, the default cut-off is indicated by a square; the performance of a random classifier is included. (**C**) As in (**A**) for the yeast dataset. (**D**) as in (**A**) for the mouse dataset. (**E**) as in (A) for the human dataset. (**F**) as in (B) for the yeast dataset. (**G**) as in (**B**) for the mouse dataset. (**H**) as in (**B**) for the human dataset.

We determined the precision and recall for each tool using each tool’s documented default settings. We also calculated the F1 score to capture the collective impact of both metrics. In the oligo dataset, the precision was high (>0.75) for all tools, while they returned either very high or very low recall F1 score (Figure 3A). In particular, the multi-sample tools performed better than the single-sample tools, except for EpiNano-SVM. We then generated precision-recall curves by considering various significance cut-offs in addition to the default setting, as described in the Methods (Figure 3B). Even with very different settings the performance of the tools was always good in terms of precision, while it recapitulated the preference for low or high recall obtained at default settings. Tools performed similarly also in terms of Area Under the Precision-Recall Curve (AUPRC), with a high fraction of overlapping AUPRCs 95% Confidence Intervals (Supplementary Figure S2, see Supplementary Data available online at http://bib.oxfordjournals.org/). This is due to the small number of positive bins (1543) which resulted in large CIs compared to the range covered by the AUPRCs (the median CI covered 27% of the range). Noticeably, this resulted in the overlap of four tools with the random classifier whose AUPRC was inflated due to the high fraction of positive bins.

For the yeast, mouse and human datasets, we integrated various orthogonal methods based on short reads sequencing for the profiling of m6A as surrogate of the missing ground truth: m6A-seq, MAZTER-seq, miCLIP2 and GLORI (as outlined in Figure 1B and detailed in the Supplementary Data, see Supplementary Data available online at http://bib.oxfordjournals.org/). The performance of all tested tools in these datasets was significantly worse than the oligos dataset, especially in terms of recall. None of the tools were able to obtain both high precision and high recall using the default settings. Furthermore, the tool’s performance for precision and recall was different among the three non-synthetic datasets, especially comparing mammalian with yeast datasets (Figure 3C-E). The precision-recall curves generalized this trend (Figure 3F-H). Indeed, the yeast dataset presented large AUPRC CIs due to the limited number of positive bins (1453), while the opposite was true in mESC and HEK293T (84 060 and 18 932 positive bins respectively); this resulted in a variable fraction of overlapping tools (Supplementary Figure S2, see Supplementary Data available online at http://bib.oxfordjournals.org/). In the latter datasets, the overlaps were more frequent when considering smaller bin sets (RRACH+ and/or high-coverage bins). Nevertheless, only Tombo CIs consistently overlapped with those of Nanocompore (in yeast) and Yanocomp (in human). Noticeably, the best performing tool for each analysis usually did not overlap with others independently from the number of positive bins. The tuning of the significance cut-off allowed exploring the precision-recall space, penalizing one metric in favour of increased performance for the other.

### Performance at RRACH and for highly expressed sites

The analysis presented above ignores three key features of m6A, which distinguish the yeast, mouse and human datasets from the oligos one: the existence of preferred sequence contexts, the existence of exclusion zones where m6A is unlikely to be deposited, and the *in vivo* stoichiometry of the marks. Indeed, m6A is preferentially found at RRACH sequence motifs [4], and typically has low prevalence and stoichiometry [36]. In addition, it has recently been found to be markedly excluded from extreme transcripts ends and from the regions adjacent to exon–intron junctions [37]. Therefore, we tested whether restricting the analyses to those bins containing RRACH sequence motifs, falling outside of exclusion zones, or having substantial expression would improve the performance of the considered tools.

Reassessing the mouse precision-recall curves in the context of the RRACH containing bins only marginally improved the tools performance in terms of precision (compare Figure A and B with Figure 3D and G). Re-evaluating the tools only for those bins that are outside exclusion zones did not significantly contribute to increasing the metrics (Figure C and D). Rather, imposing a filter on expression markedly improved the performance, especially in terms of recall (Figure E and F). The positive impact of the filter on expression was confirmed in human (Supplementary Figure S3, see Supplementary Data available online at http://bib.oxfordjournals.org/).

**Figure 4:**
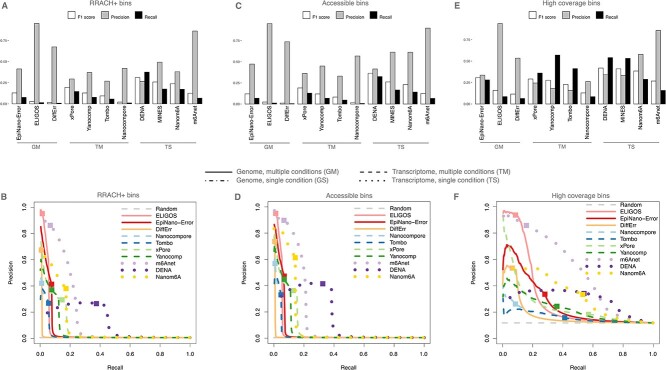
Agreement with reference sets of m6A hits on RRACH+, accessible, and high-coverage bins. (**A**) Precision, recall and F1 score for each tool executed at default conditions on the mouse dataset on RRACH+ bins. According to Supplementary Table 1, GM and TM identify tools working on the genome (G) or transcriptome (T) space and requiring multiple conditions, respectively. GS and TS identify tools working on the genome (G) or transcriptome (T) space and requiring a single condition, respectively. (**B**) Precision and recall curves at different cut-off values for the tools indicated in (A) on the mouse dataset; for each tool, the default cut-off is indicated by a square; the performance of a random classifier is included. (**C**) as in (**A**) for DRACH+ bins outside of splice-site exclusion zones. (**D**) as in (**B**) for DRACH+ bins outside of splice-site exclusion zones. (**E**) as in (**A**) for bins with high coverage. (F) as in (B) for bins with high coverage.

### Sequence features associated to true positive, false positive and false negative hits

We then asked whether there are specific sequence features where m6A marks are particularly easier or more difficult to detect. For each tool, we tested if there are sequence features that are enriched within either true positive (TP) or false positive (FP) bins.

**Figure 5:**
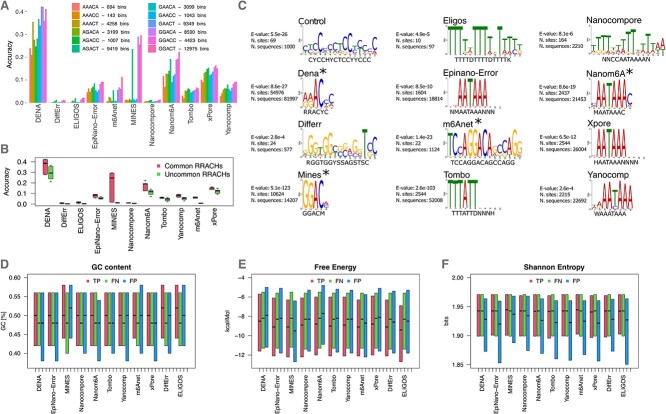
Sequence features associated with true positive, false positive and false negative hits. (**A**) m6A hits of each tool were stratified based on their association to specific RRACH motifs, and their number and accuracy on the mouse dataset was reported. (**B**) Distribution of accuracy stratified for common and uncommon RRACH motifs. (**C**) De novo motif enrichment analysis was performed on 50 nt regions centred at false positive hits for each tool on the mouse dataset, and the most significant motif was reported, together with statistical significance and consensus motif; tools marked with ^*^ are restricted to RRACH/DRACH motifs by implementation. (**D**) Distribution of the GC content for 50 nt regions centred at true positive (TP), false negative (FN) and false positive (FP) m6A hits. (**E**) as (**D**) for the free energy. (**F**) as (**D**) for the Shannon entropy.

We determined for each tool the accuracy of all the 12 variants of the RRACH motif, finding up to several fold differences (Figure 5A). Interestingly, the most common variants—those that are more often associated with m6A marks *in vivo*—are also those with the highest accuracy for all the tools (Figure 5B). Regarding the false positive bins, we searched for enriched motifs compared to a shuffled background (Figure 5C). We recapitulated the expected AC pattern only for the tools which constitutively analyse RRACH sites. For the other software, we obtained repetitive sequences mainly enriched in Ts and As which did not match with any known m6A motif.

We also characterized TP, FP and false negative (FN) bins in terms of GC content, free energy, which are proxy for the transcripts structural complexity, and Shannon Entropy, which is indicative of sequence information content. For all the tools we observed lower GC content and higher free energy in FN bins compared to TP ones, suggesting that all methods tend to miss m6A hits in less structured sequence contexts (Figure 5D and E). Rather, FP bins exhibited lower and more heterogeneous Shannon entropy compared to the other two classes (Figure 5F). This observation is in agreement with the repetitive motifs that we observed for tools not restricted to RRACHs, suggesting that FP in Nanopore based methods tend to occur in low complexity regions.

### Saturation analysis

**Figure 6:**
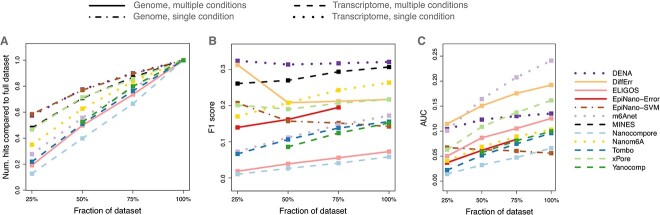
m6A calling saturation analysis. (**A**) Saturation analysis for m6A calling by various tools on the human dataset; the number of hits (y-axis) identified on subsets of the whole dataset (x-axis) is reported as a proportion of the number of hits identified on the whole dataset. (**B**) As in (**A**) where the y-axis reports the corresponding F1 score. (**C**) as in (**A**) where the y-axis reports the AUPRC.

Finally, we exploited the high coverage of the in house sequenced human dataset to thoroughly assess the impact of coverage depth on the tools’ performances. Indeed, while it is clear that higher coverage is beneficial for a more comprehensive identification of m6A sites, it is unclear, at the considered sequencing coverage, how closely we are reaching the saturation of m6A calling. To this end, we determined the number of m6A hits identified by each tool on 25%, 50% and 75% of the reads for the human dataset and compared it to the number of hits called on the entire dataset. We observed an increase in the number of hits with higher coverage for all the tools (Figure 6A) —with the exception of DiffErr, which showed the opposite trend. We then evaluated the impact of sequencing depth on the F1 score for each tool’s default conditions and observed marginally improved performances with higher sequencing coverage, except for EpiNano-SVM and DiffErr, which showed an opposite trend (Figure 6B and Supplementary Figure S4, see Supplementary Data available online at http://bib.oxfordjournals.org/, respectively). Finally, when exploring precision and recall values obtained varying the confidence parameter, we noticed a consistent increase in AUPRC with higher sequencing coverage, except for EpiNano-SVM (Figure 6C). Altogether, these results indicated that, while with the coverage of a PromethION flow-cell we are getting closer to saturation, additional sequencing would be likely beneficial and lead to the identification of additional m6A sites.

### Replicates merging

For all the analyses discussed so far, replicates were presented as separate samples to the methods designed to handle them; in agreement with the specifications of the developers. To address this aspect, we focused on the tools capable of replicates analysis, and we reanalyzed the yeast dataset by providing either replicates as separate samples or combining them. Most of the tools involved in this analysis were indeed affected by the merging of replicates (Supplementary Figure S5, see Supplementary Data available online at http://bib.oxfordjournals.org/). While the two configurations typically differed in terms of number of significant sites, the number of replicate samples was not predictive of the number of identified sites. However, the hits of the configuration with the smaller set of methylated sites were typically a subset of the other configuration (overlap always greater than 59%, and over 99% for four out of six tools).

## DISCUSSION

The advent of Nanopore sequencing of native transcripts generated rich datasets whose potential is still being explored. Numerous computational methods were developed for the analysis of these data, converting ionic current features into valuable information regarding RNA sequence, splicing variants, structure and polyA tails. These data promised to be highly informative on the multitude of modifications that decorate coding and non-coding transcripts. Indeed, numerous methods were published in a few years to profile the epitranscriptional landscape from dRNA-seq data, and others are likely to be available soon. However, users are left with limited guidance on how to prioritize the choice of the tool.

We benchmarked 14 tools—all those that were available in November 2022—for the detection of m6A on these data (Supplementary Table 1, see Supplementary Data available online at http://bib.oxfordjournals.org/). We applied them to the analysis of four different dRNA-seq datasets with specific strengths and limitations (Figure 1). The oligos dataset represents an artificial condition that poorly recapitulates the relative location and frequency of *in vivo* marks, and that lacks the confounding effect of additional modifications that might be present in proximity of m6A marks—even though in a yet unknown manner. However, as an important advantage, the ground truth in terms of m6A positioning and abundance is known in the oligos dataset. The yeast, mouse and human datasets lack a ground truth, but fully represent *in vivo* conditions of location, stoichiometry and context of m6A marks. While the yeast transcriptome is compact, and sequenced at high depth, the mouse transcriptome is significantly larger but sequenced at lower depth. To complement these datasets, we sequenced a human dataset taking advantage of the higher throughput PromethION platform, which could provide dRNA-seq of a transcriptome of complexity comparable to the mouse’s, but at a higher sequencing depth.

The datasets processed for this work were heavy and complex, consisting of >22 M files totalling 4 TB of raw data, and likewise is the effort required for their analysis. We experienced difficulties completing the run for several tools, and for few of them we had to renounce, due to lack of evident progress in the run or the unexpected generation of empty output files. The tools returned a remarkably different number of hits. However, these had a plausible distribution, given the expected location in each specific test datasets, and pairwise overlap (Figure 2). These results suggested that the tools might have different performance in terms of precision and recall.

The m6A calls returned by the tools were compared to reference sets of m6A hits (Figure 1) to define precision, recall and F1 scores (Figure 3). In the case of the oligos dataset, the reference set is known by design and includes all the As available in the artificial sequences. For yeast, mouse and human, the reference set was obtained by integrating various independent datasets obtained with recent leading approaches for m6A profiling relying on short reads high-throughput sequencing. The analysis of precision and recall indicated that the tools performed very well on the synthetic oligos dataset, while the yeast, mouse and human datasets represented a more challenging task. The precision versus recall curves showed that the default settings for some of the tools nicely identify a good trade-off between precision and recall. For other tools, these curves could be used to point to better cut-off conditions for those users aiming at maximizing both metrics. Altogether, these analyses showed that, if needed, there is broad space to steer the preference towards either one of the two metrics.

The low F1 scores in the yeast, mouse, and human datasets compared to the oligos dataset can possibly be attributed to the stoichiometry of m6A marks at each position or differences in bias between the orthogonal reference m6A sets and nanopore based m6A techniques. By mixing unmodified reads and modified reads from the oligos datasets, we could simulate a more biologically equivalent m6A stoichiometry in the synthetic oligos to address its effect on nanopore m6A detection. Most of the software tools had lower F1 scores at lower m6A stoichiometry in the *in silico* mixed samples (Supplementary Figure S6, see Supplementary Data available online at http://bib.oxfordjournals.org/), which agrees with previous observations using a subset of the tools we tested with NanOlympicsMod (Nanocompore and xPore). This suggests that the lower expected m6A stoichiometry in the biological datasets is at least partially responsible for the observed reduced F1 scores.

The tested tools can be grouped according to the genome or the transcriptome being the required reference sequence, and according to their requirement or not of a baseline sample depleted of the modification of interest (Supplementary Table 1, see Supplementary Data available online at http://bib.oxfordjournals.org/). Tools of any class performed well with the synthetic oligos dataset, especially in terms of precision, while at default settings they had different preferences in terms of recall, the multi-sample tools typically achieving higher recall values. The high precision obtained by all tools in this dataset is also a consequence of the high density of m6A nucleotides, constraining the minimum precision value to the ratio of m6A+ bins to all the bins. The multi-sample tools, such as ELIGOS, Yanocomp, DiffErr and Nanocompore performed better in the yeast dataset, probably benefiting from the higher coverage. Rather, the single-sample tools working in transcriptome space, such as m6Anet and DENA, performed better in the mouse and human datasets, likely because they were able to capture m6A features in complex transcriptomes. Interestingly, m6Anet and DENA were applied for the analysis of yeast and mouse datasets despite they were trained on data obtained for *Homo sapiens* and *Arabidopsis thaliana* species (Supplementary Table 1, see Supplementary Data available online at http://bib.oxfordjournals.org/), which might partially explain those tools’ reduced performance in the yeast dataset. Similarly, EpiNano-SVM and Nanom6A were trained on synthetic oligos which lack the complexity of real transcriptomes and potential confounding factors like endogenous RNA modifications, potentially impacting their performances when applied to yeast, mouse and human data.

We tested whether restricting to specific sets of bins, such as those associated with RRACH motifs, high coverage or far from exclusion zones, could improve the performance (Figure :). We found that, for those tools that are limited by design to only test RRACH or DRACH sites, this reassessment had limited chances of significant improvement. Only by filtering for highly expressed bins significatively boosted precision and recall.

Finally, we identified specific sequence features that characterized TP, FP and FN bins (Figure 5). We showed that m6A at specific RRACH variants can be detected with higher accuracy and that the tested tools differ in terms of sequence motifs that could divert them. Additionally, we revealed that all the considered Nanopore-based tools tend to miss hits in unstructured regions, while identifying m6A unsupported by orthogonal techniques in low complexity domains. These observations are indicative of sequencing platforms-specific biases. For instance, the systematic occurrence of FNs in low-entropy bins could stem from the suboptimal performance of dRNA-seq with homopolymers, or from short reads alignment issues in repetitive sequences. Furthermore, short-read based methods may exhibit biases towards less structured transcripts, where antibodies and chemical compounds could more efficiently access the substrate. Further in-depth studies are warranted, likely with both short-read and Nanopore based approaches, to better understand and assess these limitations.

We then evaluated the tools’ performances in terms of number of hits, F1 score at default conditions and area under the PR curve at different coverage depth values. Results from this saturation analysis showed a marked increase in the number of hits at higher coverage depth, with a decrease in the slope of the curve between 75% and 100% of the available coverage, suggesting the curve was about to reach a plateau. Interestingly, DiffErr showed an opposite trend, suggesting that the tool may be designed to use the additional information to restrict the set of candidate hits. When considering the F1 score value at default conditions, we observed only marginally improving performances with higher sequencing coverage for most of the tools. This result may suggest that the increase in coverage depth leads to an increase in the number of hits which, in turn, results in a higher sensitivity; however, the increase in sensitivity is also accompanied by a decrease in precision, with an overall effect of saturation of the F1 score. Finally, most of the tools showed an increase in the AUPRC with higher coverage depth, consistent with an overall improvement in m6A identification with higher coverage. This result also suggests that cut-off settings should be tuned also considering the available coverage depth. Only EpiNano-SVM showed a decrease in the AUPRC value at higher coverage depth: this result may be explained by features of the dataset used for the training of the algorithm. Interestingly, we showed that tools based on differential errors identification, as ELIGOS and DiffErr, were able to identify a consistent number of hits also in both the mouse and human datasets, confirming the presence of a differential error due to the presence/lack of m6A, despite the different sequencing platform and the associated base-calling model.

In general, we recommend focusing on high-coverage regions, as this allows obtaining a marked increase in the tools’ performances. We observed that m6Anet outperformed all the tools both in the mouse and in the human dataset. On the oligos and yeast datasets, m6Anet was not among the top performers, possibly due to the fact that we were using the default neural network trained on a human dataset. If it is not possible to re-train m6Anet on a dataset from a related species, we advise the users to run multi-sample tools for yeast, such as ELIGOS and Nanocompore, as they were the top performers in terms of AUC in yeast and oligos datasets. As an alternative, we advise the users to integrate the predictions from multiple tools for obtaining a more accurate set of m6A modifications. The set of tools may be chosen by ranking them by the AUC value obtained in this study. In particular, the user may want to build a meta-classifier integrating the performances of multiple tools. We picked the top 5 performing tools, according to the AUC value, in the analysis of high-coverage bins of human dataset, and tested the performance of three meta-classifiers obtained as either the intersection, the majority vote or the union of their predictions. The majority vote classifier was the most balanced among the three in terms of recall and precision, allowing to obtain the highest F1 score at default conditions (0.40) (Supplementary Figure S7, see Supplementary Data available online at http://bib.oxfordjournals.org/), outperforming the best tool (MINES, F1 score = 0.38).

A recent study from Zhong *et al*. [34] benchmarked multiple tools for m6A detection on dRNA-seq data focusing on the mouse epitranscriptome. Our results are largely consistent with the results reported by the Zhong study. However, we would like to stress key points where our study significantly improves compared to what has been published. First, in our study we describe the development and release of NanOlympicsMod, a Nextflow pipeline exploiting containerized technology for the benchmarking of m6A detection tools on dRNA-seq. A similar resource was completely missing in the Zhong paper and will be of utmost importance in the field. Not only for reproducing the results, but also for testing further tools that are likely to be published soon in this very active research field. Second, we benchmarked 14 tools on datasets from three different species, yeast, mouse, and human. The Zhong paper was primarily focused on a mouse dataset only. Third, the production of a high sequencing depth dataset for human allowed us to perform a saturation analysis of m6A calling that was not included in the Zhong paper. Fourth, we also tested the tools on a dataset relying on synthetic oligos, which was missing in the Zhong paper. This is particularly important, since it is the only condition in which the ground truth is known, being the position of m6A known by design of the oligos.

Altogether, our analyses indicate that the target sequencing depth and the adopted cut-off settings are likely the most important choices for m6A profiling on dRNA-seq data. The choice of the specific tool likely depends also on whether one wishes to map the m6A hits directly on specific transcripts or not (genome versus transcriptome-based tools), whether one wishes to have direct evidence of the modification of interest (multi-sample versus single-sample tools) and whether m6A or other marks are sought. The NanOlympicsMod framework represents a portable, reproducible, and scalable resource to run and compare Nanopore Direct RNA Sequencing-based tools for the profiling of m6A or other marks, which will facilitate these decisions and will streamline the test of additional detection tools. Moreover, we think that the produced sequencing dataset will serve as a valuable resource for set-up and validation of novel dRNA-seq based tools.

## METHODS

### Cell culture treatment with STM2457

HEK293T cells were grown using Dulbecco's Modified Eagle Medium (DMEM) supplemented with 10% Fetal Bovine Serum (FBS) and 1% penicillin–streptomycin. Cells were treated with vehicle (Ethanol 100%) or with 20 μM STM2457 and incubated for 24 hours at 37 °C.

### RNA extraction and mRNA purification

Total RNA was extracted from 10 million cells using Qiazol (Qiagen 79306) and RNeasy Micro Kit (Qiagen 74004). mRNA purification was performed with 100 ug of Total RNA using μMACS™ mRNA Isolation Kit (Miltenyi Biotec 130–075-201) following the manufacturer’s protocol.

### Bulk m6A quantification

Bulk m6A mRNA levels were quantified using Elisa kit (EpiQuik-Epigentek). A total of 100 ng of mRNA were loaded. Samples were incubated with m6A antibody for 1 h following manufacturer’s protocol. The detected signal was quantified colorimetrically by reading the absorbance in a microplate spectrophotometer at a wavelength of 450 nm.

### Nanopore direct RNA sequencing

A total of 150 ng of mRNA were used as an input for Nanopore Direct RNA sequencing libraries preparation. A total of 152 ng and 120 ng of library were obtained for HEK293T control and HEK293T Storm, respectively. Both samples were loaded on PromethION flow cells, with 7562 pores for HEK293T control and 8233 pores for HEK293T treated with STM2457.

### Reference files and datasets preparation

See Supplemental Data for a comprehensive description of the considered and produced datasets. The description on how the data were processed follows here:

#### Synthetic oligos

The sequences of synthetic oligos were downloaded from [38] and concatenated into a single fasta file. Then, the coordinates of all ‘A’ nucleotides were obtained using vMatchPattern function of Biostrings v2.66.0 R package and saved to file in bed format.

#### Yeast dataset

The yeast Nanopore dRNA-seq data were retrieved from [39]. Reference genome and transcriptome files for SK1 yeast strain, together with the set of reference peaks, were downloaded from [39]. In particular, ‘MvO’ genome assembly was downloaded from http://cbio.mskcc.org/public/SK1_MvO/, while ‘SGD_2015_JRIH00000000’ reference transcriptome was downloaded from http://sgd-archive.yeastgenome.org/sequence/strains/.SK1/SK1_SGD_2015_JRIH00000000/. Since we could not retrieve a proper gtf annotation file for SK1 strain, we first aligned the transcriptome to the genome with minimap2 v2.24.0 [40] with -x splice mode. We then converted the alignment bam file in bed12 format with bedtools bamtobed v2.30.0 [41] and finally obtained a gtf annotation file using a combination of bedToGenePred and genePredTogtf from UCSC tools v377 (https://github.com/ucscGenomeBrowser/kent). The m6A hits reference set was obtained combining the hits from MAZTER-seq [18] and m6A-seq [42] released as supplemental material in Garcia-Campos *et al*. and Schwartz *et al*., respectively, and collapsing overlapping hits with bedtools merge v2.30.0 [41].

#### Mouse dataset

The mouse Nanopore dRNA-seq data were retrieved from [43]. Reference genome and transcriptome fasta files for mouse, together with gtf annotation file, were downloaded from https://www.gencodegenes.org/mouse/release_M23.html. The m6A reference set was obtained combining the hits from miCLIP2 [15] and GLORI [23] released as supplemental material in Körtel *et al*. and Liu *et al*. respectively, and collapsing overlapping hits with bedtools merge v2.30.0 [41].

#### Human dataset

The human dRNA-seq data were produced as part of this work and were uploaded to SRA (BioProject ID: PRJNA995902). The reference genome for human was downloaded from https://hgdownload.soe.ucsc.edu/goldenPath/hg38/bigZips/hg38.fa.gz, while the gtf annotation file was downloaded from https://ftp.ensembl.org/pub/release-109/gtf/homo_sapiens/Homo_sapiens.GRCh38.109.gtf.gz. Sequence and annotations for chromosome chr1 were then subset from the full files with bash custom scripts. The transcriptome fasta file for human was generated from the reference fasta and the annotation gtf files with bedtools getfasta v2.30.0 [41]. The m6A reference set was obtained downloading GLORI hits release as supplemental material in Liu *et al*. [23] and subsetting hits from chromosome chr1.

### The NanOlympicsMod workflow

The NanOlympicsMod workflow is composed of four steps: *pre-processing*, *tools execution*, *post-processing* and *comparative analyses* (Figure 1A).

#### Pre-processing

FAST5 files for the four datasets were re-basecalled using Guppy v6.2.1 with command ‘guppy_basecaller -i <input_path> -r -x “auto” -s <save_path> --fast5_out -c rna_r9.4.1_70bps_hac.cfg’. FAST5 files are converted from multi-reads to single-read with multi_to_single ONT API v4.0.0 (https://github.com/nanoporetech/ont_fast5_api) and the base-called sequences are extracted in FASTQ format with Poretools v0.6.0 [44]. Then, sequencing reads are aligned to the transcriptome (−x map-ont) and to the genome (−x splice) with Minimap2 v2.24.0 [40]. Alignment files are then used for signal resquiggling with both Tombo v1.5.1 [45] and Nanopolish v0.8.4 [46]. The pre-processing steps are performed in parallel for all the samples involved in the analysis, and the resulting files are then provided to each tool according to their requirements.

### Tools execution

Fourteen tools for m6A detection on dRNA-seq data are run in parallel and the corresponding output is stored in a dedicated folder tree. As outlined in Supplementary Table 1, we ran ELIGOS v2.1.0 [43], m6Anet v1.1.0 [47], MINES [48], Tombo v1.5.1 [45], DRUMMER [49], EpiNano-SVM v1.2 [38], EpiNano-Error v1.2 [38], DENA [50], Yanocomp v0.2 [51], Nanocompore v1.0.3 [39], xPore v2.0 [52], DiffErr v0.2 [29], nanom6A [53] and nanoDoc [54]. If replicates are available, they are provided as separate samples to the tools designed to handle them (Yanocomp, xPore, Nanocompore, m6Anet, ELIGOS, DiffErr and DRUMMER); otherwise they are merged.

### Post-processing

Post-processing is based on an R script that collects all the tools output. This is heterogeneous in terms of format and information and this workflow step converts each tool’s output into a common file format. First, for those tools which rely on transcriptome alignment, a lift-over from transcriptome-based to genome-based coordinates is also performed with the transcriptToGenome function from ensembldb v2.18.4 R package. Although this may not be a compulsory step in a standard analysis, it was required for comparing these tools to those providing hits in genome-based coordinates and to the reference sets. Finally, a BED file for each tool is produced, which contains the genomic position of each call, its modification status (modified or unmodified) - defined according to criteria suggested by the developers - and the numerical value which drove the classification, if available (i.e. False Discovery Rate, modification probability, *P*-value).

### Comparative analyses

The comparative analysis consists of an R script designed to process all the BED files returned by the post-processing step and to perform the analyses described in the main text. The details of these analyses are described in the section *Additional analyses*.

### Additional analyses

#### Binning

The gene space is first binned into fixed-size windows, starting from the 3′ end of the gene. In case the gene length is not multiple of the window size, the last window at the 5′ end is discarded. We chose 5 nt as window size for the oligos dataset, and 50 nt for yeast, mouse and human datasets. Smaller windows of 40, 30, 20 and 10 nt were also tested for the human dataset, showing a small while progressive increase in the AUC with increasing window size (Supplementary Figure S8, see Supplementary Data available online at http://bib.oxfordjournals.org/). The smaller window size for the oligos dataset was required by the high density of As, since a larger window size would have resulted in having only m6A+ bins.

### Collecting the m6A hits of each tool

A *matrix of m6A hits* with number of rows equal to the number of bins and number of columns equal to the number of tools plus 1 (the reference set) is created. The matrix columns are initialized to 0 or − 1, depending on whether the confidence parameter for the tool needs to be maximized (i.e. probability of modification) or minimised (i.e. *P*-value), respectively. For each tool, the overlaps between the hits and the genes bins are evaluated and the value of the confidence parameter is reported in the corresponding cell of the matrix. In case multiple hits occur within the same bin, the value corresponding to the least confident hit is reported. In case smaller confidence parameter values imply higher confidence for a tool (e.g. *P*-value), scores for that tool are multiplied by −1. This is required by PRROC v1.3.1 R package [55] that we used for plotting Precision-Recall curves, as it expects that higher values of the parameter correspond to higher confidence calls (see below).

An auxiliary *binary matrix of hits* is also created, containing 1 or 0 depending on whether the bin should be called as m6A+ or not after filtering the hits at the default parameter threshold. This matrix is then used for evaluating the hits’ overlaps and for evaluating recall, precision and F1 score at default conditions.

### Overlap analyses

The overlap of m6A hits for each pair of tools is stored in a matrix with the number of rows and columns equal to the number of tools. Each (*i,j*) cell of this matrix reports the number of m6A+ bins identified by both tools *i* and *j*, divided by the number of m6A+ bins identified by tool *i* (the one reported in the rows in Figure 3). The matrix was then visualised as a heatmap with the pheatmap function of pheatmap v1.0.12 R package.

### Comparison of hits to reference m6A set

The tools default conditions were defined as described in Supplementary Table 4, see Supplementary Data available online at http://bib.oxfordjournals.org/. The *binary matrix* of m6A hits at default conditions was used to compare the bins identified as m6A+ for a given tool to the bins classified as m6A+ according to the reference set of each dataset. The recall was then determined as the ratio of m6A+ bins in the reference set that were identified as m6A+ also by the tool, while the precision was computed as the ratio of m6A+ bins identified by the tool that were confirmed by the reference set. The F1 score was determined as the harmonic mean of precision and recall.

For assessing precision and recall at various stringency cut-offs, the scores of the *matrix of m6A hits* were screened according to each cut-off and compared to the hits of the corresponding reference m6A set to define true and false positive bins for each tool. Precision-Recall curves were plotted with the *pr.curve* function of PRROC v1.3.1 R package [55] which also provided the corresponding AUPRC. The approach described in [56] was applied to estimate AUPRCs 95% Confidence Intervals.

### Selection of RRACH, accessible, and high-coverage bins

*RRACH-containing bins* were determined using the vMatchPattern function of Biostrings v2.66.0 R package.

*Accessible bins* were identified as those bins overlapping to ‘GGACC’, ‘AGACA’, ‘TGACT’, ‘AGACT’, ‘GAACT’, ‘GGACA’ and ‘GGACT’ motifs, occurring outside of inaccessible regions, defined as 100 nt at the ends of each exon, using a combination of vMatchPattern and resize function of GenomicFeatures package.

*High-coverage bins* were determined importing genome alignment files in R v4.2.1 using readGAlignments function of GenomicAlignments v1.32.1 R package and evaluating the read counts for each exon, using a combination of makeTxDbFromGFF, exons and findOverlaps functions of GenomicFeatures v1.48.3 package. The genomic coordinates of exons with more than 100 read counts were saved to a file in BED format and were used to identify the subset of high-coverage bins.

### Metagene plots

Starting from BED files including genomic coordinates of m6A hits filtered at default parameter values, we produced metagene plots showing m6A hits distribution along transcriptional units using GuitarPlot function of Guitar v2.12.0 package.

### Sequence features associated with either true or false positive calls

We first annotated genomic bins with a specific motif, in case a single hit in the reference set overlapped to the bin and to a RRACH motif. We then evaluated the accuracy (i.e. the recall) of each tool at detecting true positive bins for each motif and produced a barplot. Moreover, we performed a *de novo* motif enrichment analysis on the sequence of false positive bins using XSTREME program from MEME Suite v5.5.2 [57] (online implementation). The gene sequence of 50 nt bins where the tools detected m6A hits that were not confirmed by the reference set was extracted and used for a motif enrichment analysis, using shuffled input sequences as control. Only the top motif for each tool was reported, together with its statistical significance and the resulting consensus motif. We additionally obtained the sequences of false negative bins for each tool, which are bins not called by the tool but present in the reference set. For each tool and bin in the true positive, false negative, and false positive sets, we calculated the GC content using R, we determined the free energy using RNAfold v2.5.0 from the Vienna RNA package [58], and we calculated the Shannon entropy using the Entropy method from the R package DescTools (v0.99.49).

### Source code

The source code for the NanOlympicsMod workflow, and for reproducing all the results included in this study, are available at the following GitHub repository: https://github.com/mfurla/NanOlympicsMod.

**Key Points**

- Nanopore direct RNA-seq sequencing (dRNA-seq) allows the identification of various RNA modifications including N6-Methyladenosine (m6A)
- Numerous tools were developed to identify m6A from dRNA-seq data, however a comprehensive benchmarking across species and against established orthogonal methods is missing
- We developed the NanOlympicsMod workflow to facilitate comparing tools for m6A detection on dRNA-seq data, and we used it to benchmark 14 software on synthetic RNA oligos, yeast, mouse and human transcriptomes
- The performance of the tools varies between synthetic and real datasets and is particularly sensitive to the expression of the tested regions
- Tools relying on specific approaches, i.e. working on transcriptome or genome space and requiring single or multiple conditions, have different preferences in terms of precision and recall

## Supplementary Material

Maestri_SupplementaryData_bbae001

## Data Availability

Human Nanopore sequencing data generated as part of this study are available in SRA (BioProject ID: PRJNA995902). Oligos, Yeast and Mouse Nanopore sequencing data were derived from sources in the public domain as indicated in Supplementary Table 2.The source code for the NanOlympicsMod workflow, and for reproducing all the results included in this study, are available at the following GitHub repository: https://github.com/mfurla/NanOlympicsMod.

## Notes

**Simone Maestri** is an engineer expert in bioinformatics and Nanopore sequencing data. He was computational postdoc in Pelizzola’s group at the Istituto Italiano di Tecnologia. He moved at the University of Milano, working on the analysis of Nanopore sequencing data in the Lab of Stem Cell Biology and Pharmacology of Neurodegenerative Diseases.

**Mattia Furlan** is a physicist expert in bioinformatics, transcriptional regulation, RNA modifications and Nanopore sequencing. He works as computational postdoc in Pelizzola’s group at the Istituto Italiano di Tecnologia.

**Logan Mulroney** is expert in bioinformatics, RNA modifications and Nanopore sequencing. He has been joint postdoc with European Bioinformatic Institute and Istituto Italiano di Tecnologia. He now works as computational postoc in Nicassio’s group at the Istituto Italiano di Tecnologia.

**Lucia Coscujuela Tarrero** is expert in cancer RNA biology, RNA modifications and genomics. He works as postdoc in Pelizzola’s group at the Istituto Italiano di Tecnologia.

**Camilla Ugolini** is a physicist expert in bioinformatics, RNA modifications and Nanopore sequencing. He works as PhD student in Nicassio’s group at the Istituto Italiano di Tecnologia.

**Fabio Dalla Pozza** worked as bioinformatician in Pelizzola’s group at the Istituto Italiano di Tecnologia.

**Tommaso Leonardi** is expert in bioinformatics, RNA modifications and Nanopore sequencing. He worked as Researcher at the Istituto Italiano di Tecnologia.

**Ewan Birney** is Deputy Director General of EMBL. He is also Director of EMBL-EBI with Dr Rolf Apweiler. He runs a research group studying between-individual differences in both Japanese rice fish (Medaka) and humans, and exploring novel algorithms in sequence methods, in particular around nanopore technology.

**Francesco Nicassio** is Senior Researcher at the Istituto Italiano di Tecnologia, Principal Investigator of the Genomic Science research line. Scientific interests are centered on the exploitation of genomic approaches to the study of mechanisms in control of gene expression dynamics provided by non-coding RNAs (microRNAs and long noncoding RNAs) and their impact on cell behaviour and human disease, with emphasis on Cancer.

**Mattia Pelizzola** is a computational biologist with background in biotechnology affiliated at the Istituto Italiano di Tecnologia and Associate Professor at the Dept. of Biotechnology and Biosciences, Milano-Bicocca University. He leads a research group studying how co- and post- transcriptional events shape gene expression programs. His group employs an interdisciplinary approach combining cutting edge experimental and computational methods, including the profiling of nascent and modified RNA, single-molecule Nanopore sequencing, and mathematical modelling.
