# Supplementary material for: Benchmarking of computational methods for m6A profiling with Nanopore direct RNA sequencing
Source: Brief Bioinform. 2024 Jan 26;25(2):bbae001. doi: 10.1093/bib/bbae001 (PMC10818168; doi:10.1093/bib/bbae001)
Supplement: Maestri_SupplementaryData_bbae001 [file maestri_supplementarydata_bbae001.docx]

**Benchmarking of computational methods for m6A profiling with Nanopore direct RNA sequencing – Supplementary Data**
Simone Maestri^1,5^, Mattia Furlan^1,5^, Logan Mulroney^1,2,3,5^, Lucia Coscujuela Tarrero^1^, Camilla Ugolini^1^, Fabio Dalla Pozza^1^, Tommaso Leonardi^1^, Ewan Birney^2^, Francesco Nicassio^1,*^, Mattia Pelizzola^1,4,*^


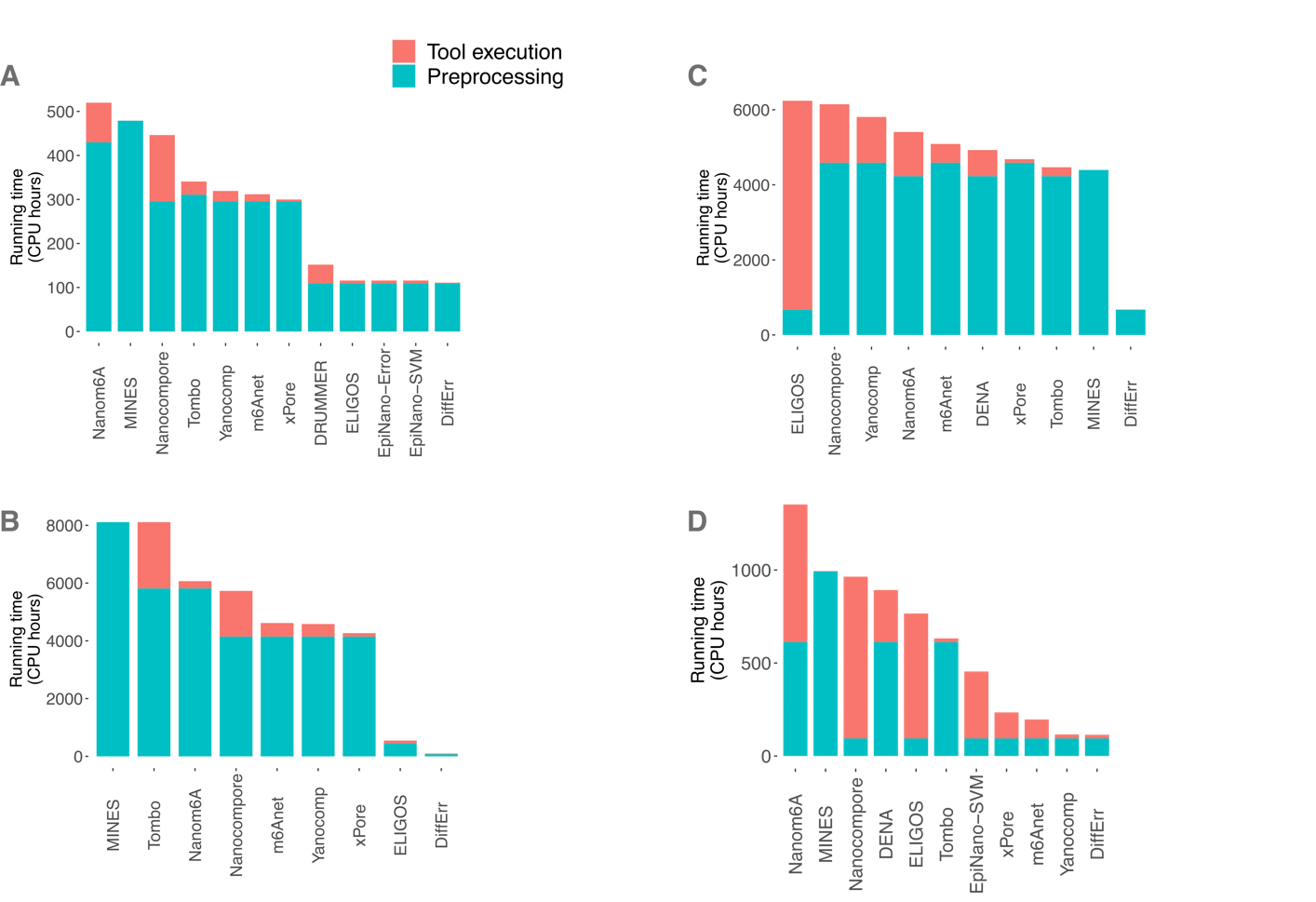


**Supplementary Figure S1 - Tools running time.** (**A**) The total running time for the oligos dataset is reported. The contribution to the total amount of time from the pre-processing steps of the NanOlympicsMod workflow and from the tools themselves is depicted in different colours. (**B**) as in (A) for the yeast dataset. (**C**) as in (A) for the mouse dataset. (**D**) as in (A) for chr1 of the human dataset.

**
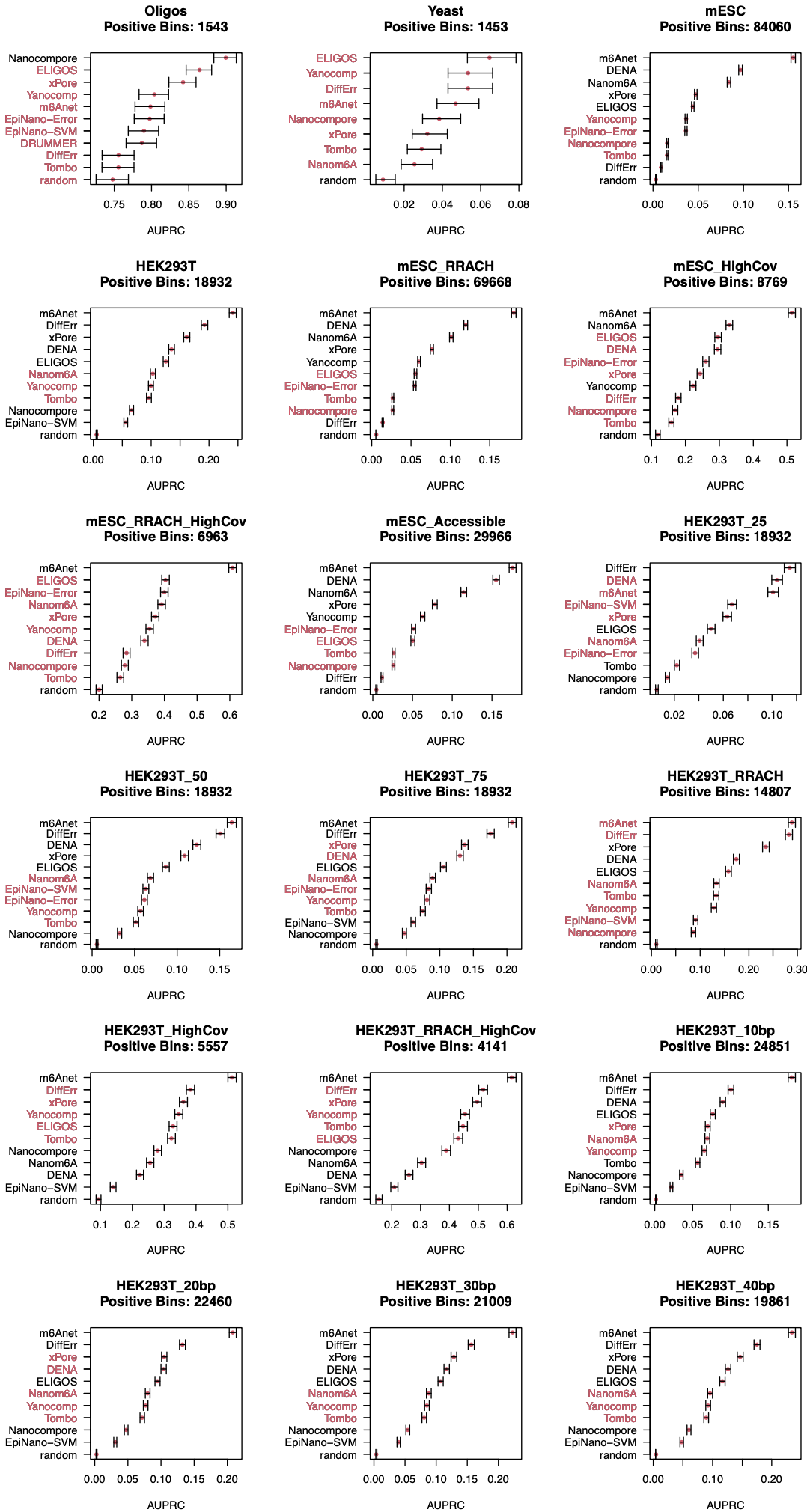
**

**Supplementary Figure S2 – AUPRC rank-plots.** Rank-plot of the Areas Under the Curve for all the Precision-Recall analyses performed. For each tool and configuration, the AUPRC value (red dot) and the corresponding 95% Confidence Intervals are reported. Tools whose Confidence Intervals overlap with others are highlighted in red. The number of positive bins for each configuration is reported in the title of the corresponding panel.


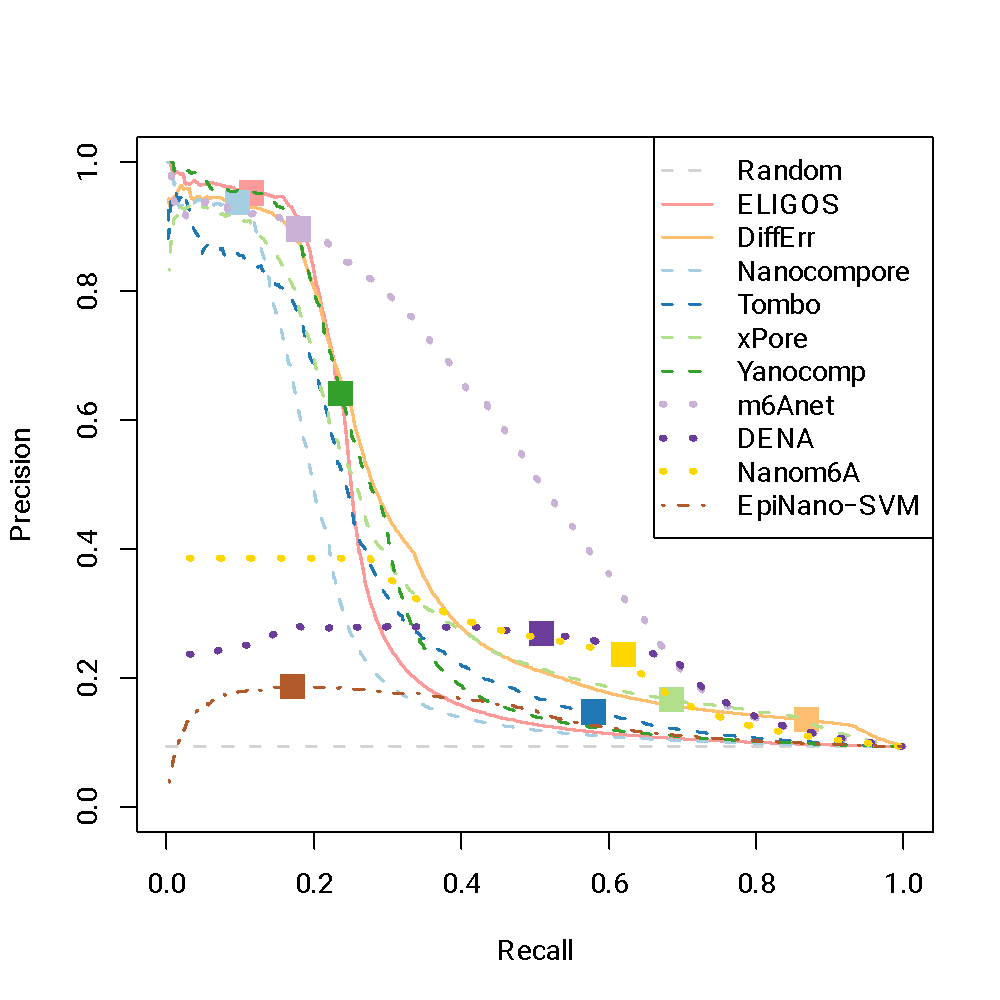


**Supplementary Figure S3 – Human precision and recall curves for high coverage bins.** Precision and recall curves at different cutoff values for bins with high coverage on the human dataset; for each tool, the default cutoff is indicated by a square; the performance of a random classifier is included.


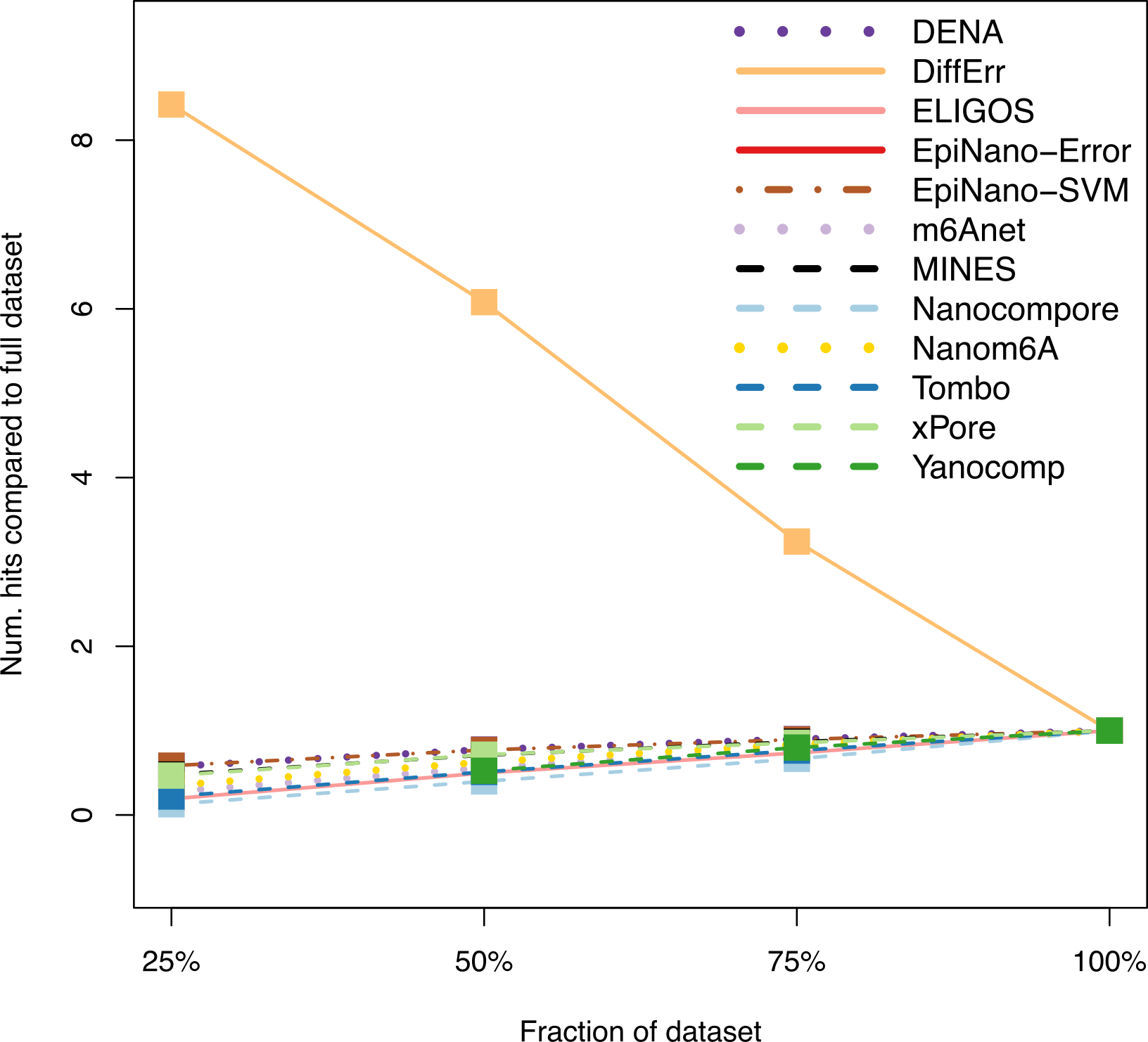


**Supplementary Figure S4 – m6A calling saturation analysis including DiffErr.** Saturation analysis for m6A calling by various tools on the human dataset; the number of hits (y-axis) identified on subsets of the whole dataset (x-axis) is reported as a proportion of the number of hits identified on the whole dataset. For DiffErr, while as expected the number of tested sites increases with the fraction of dataset, the number of hits decreases.


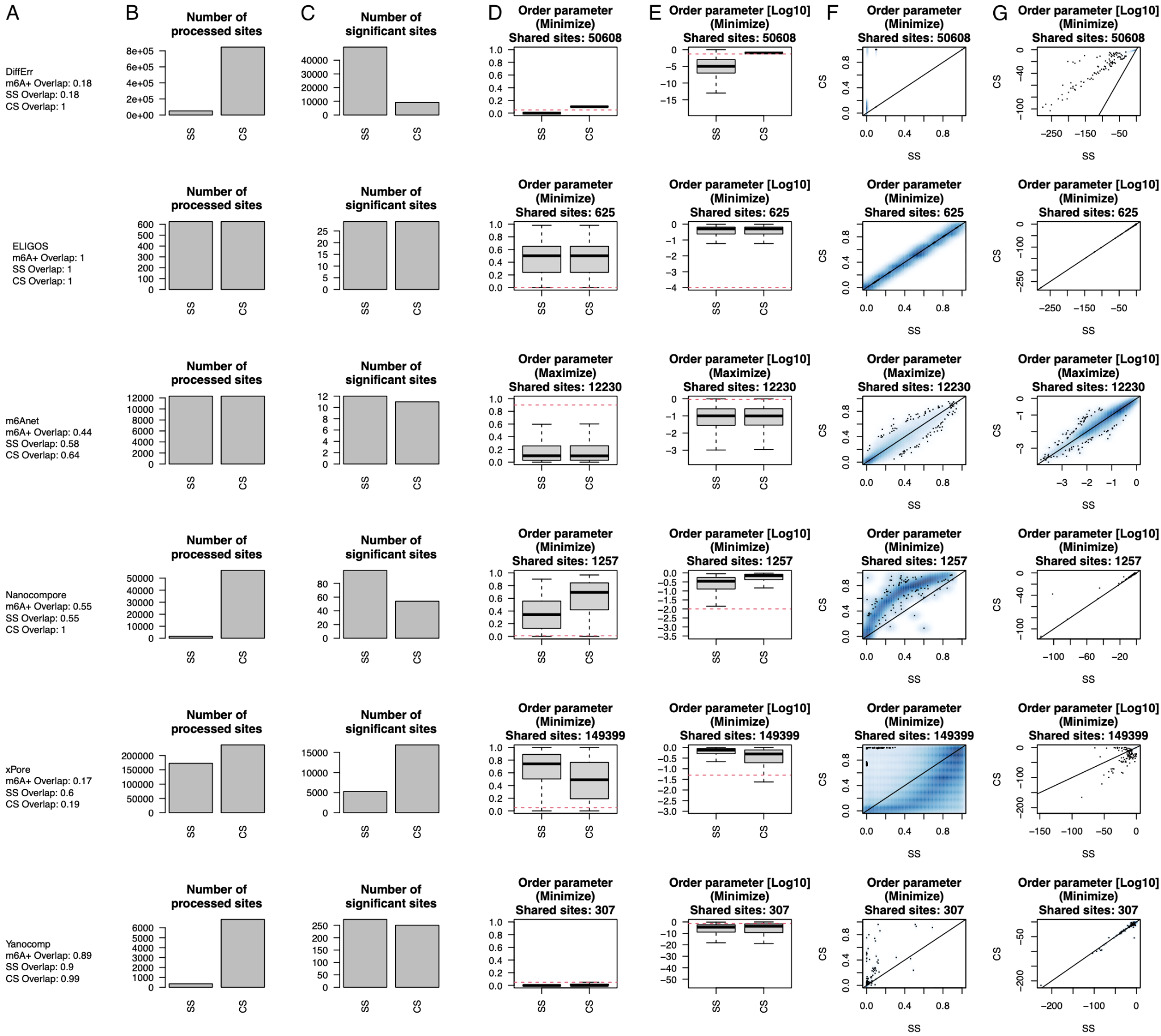


**Supplementary Figure S5 – Separate vs Combined Samples analysis.** Results comparison for tools designed to handle replicates run with Separate and Combined Samples (SS and CS respectively). (A) For each tool, we report the ratio between the number of sites classified as methylated in both the analyses, and the number of methylated sites in: at least one analysis (m6A+ Overlap), the Separate Samples analysis (SS Overlap), and the Combined Samples analysis (CS Overlap). (B) For each tool, we report the number of processed sites in the two analyses. (C) As D for methylated sites. (D) For each tool, we report the distributions of the order parameter which determines the classification of the sites. This analysis is performed on the sites processed in both the analyses (numerosity reported in the box title); the default threshold is depicted in grey. (E) As D in Log10 space. (F) Scatterplot comparing, for each tool, the order parameter which determines the classification of the sites; the bisector of the first quadrant is reported in black. (G) As F in Log10 space.

**
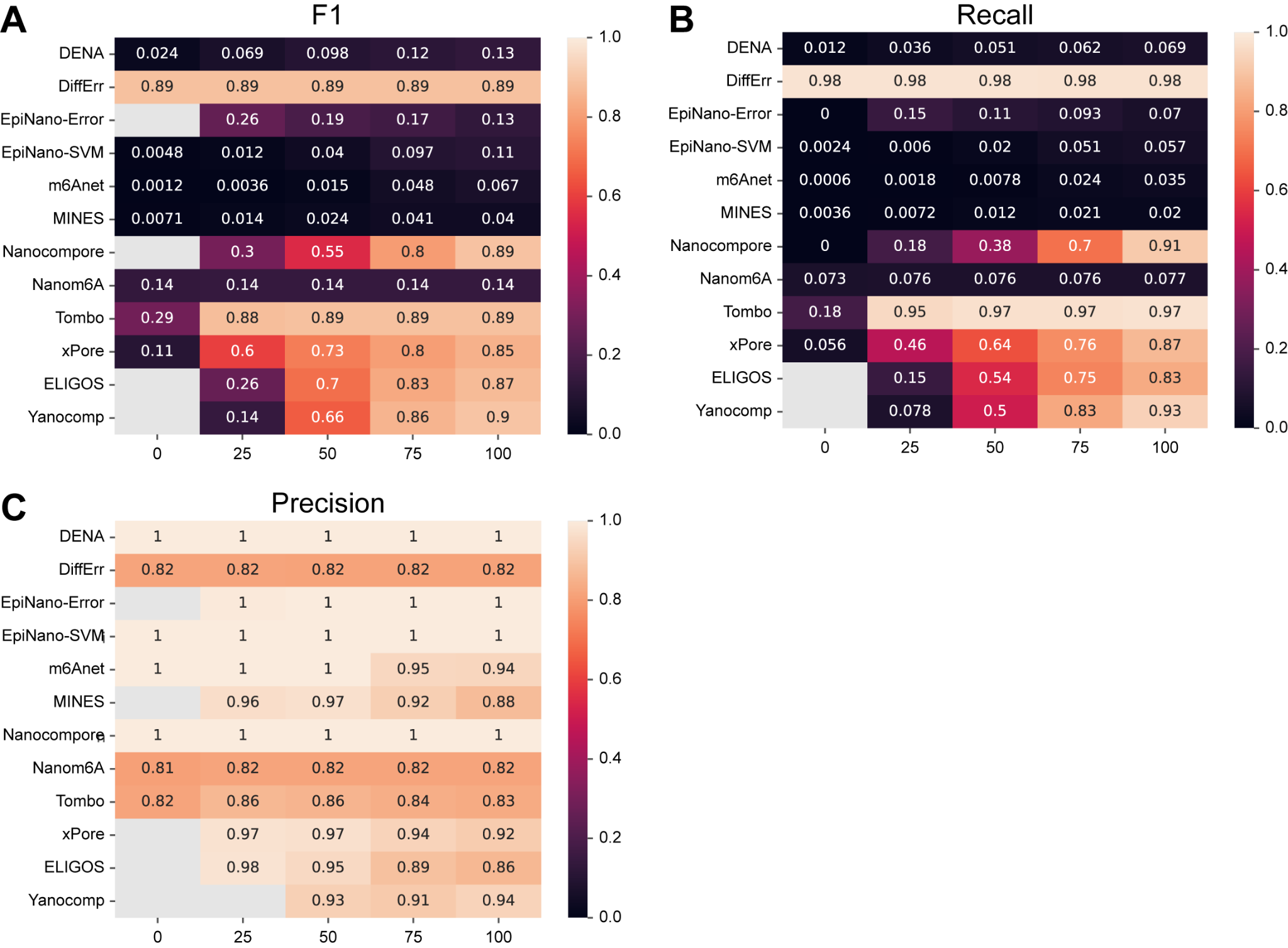
**

**Supplementary Figure S6 - Benchmarking NanOlympicsMod tools with simulated modification stoichiometry.** Unmodified and modified fast5 files from the synthetic oligos datasets were mixed in ratios resulting in 0%, 25%, 50%, 75%, and 100% m6A stoichiometry for the test sample. The reference sample was 0% modified reads which were not included in the mixed test samples. Displayed are heatmaps for the performance evaluated by F1 score (A), recall (B), and precision (C) of each tool at the five stoichiometry percentages.


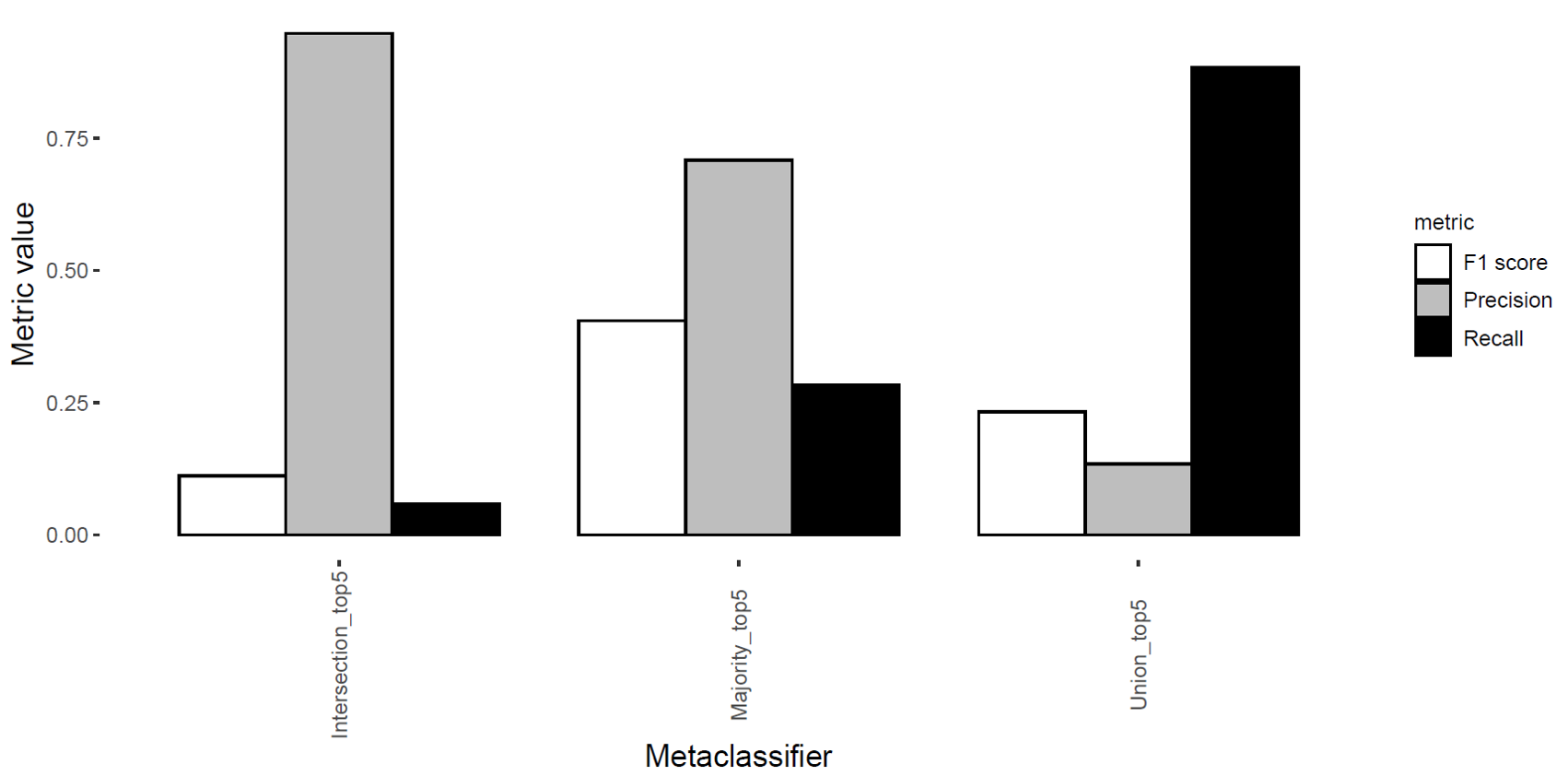


**Supplementary Figure S7 - Performances of meta-classifiers in high-coverage bins of human dataset.** Meta-classifiers obtained by doing the intersection, majority voting or union of hits at default conditions, considering the top 5 tools according to the AUC value on the same dataset, are reported. For each meta-classifier, F1 score, Precision and Recall are reported.


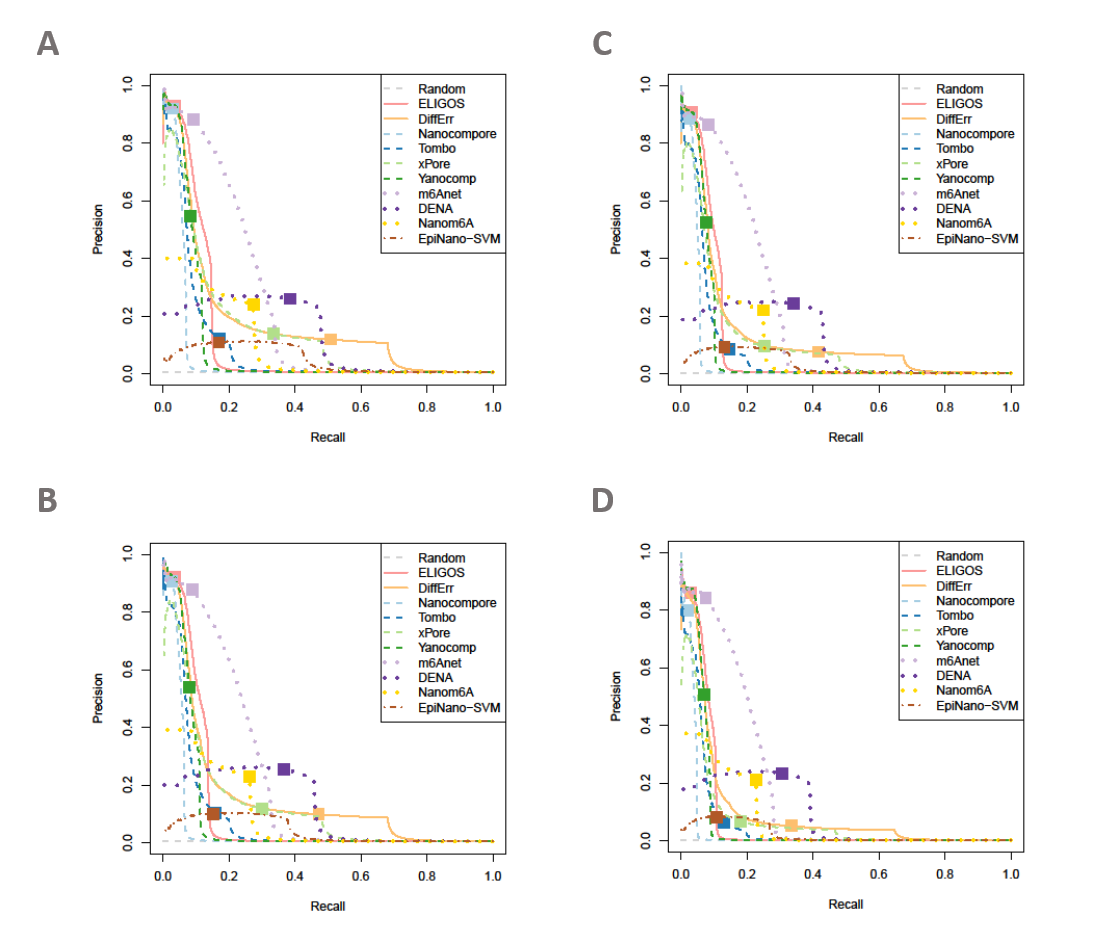


**Supplementary Figure S8 - Impact of smaller bin size on PR curves.** PR curves for human dataset obtained reducing the bin size are reported. (A) PR curves for bin size of 40 nt. (B) PR curves for bin size of 30 nt (C) PR curves for bin size of 20 nt. (D) PR curves for bin size of 10 nt.


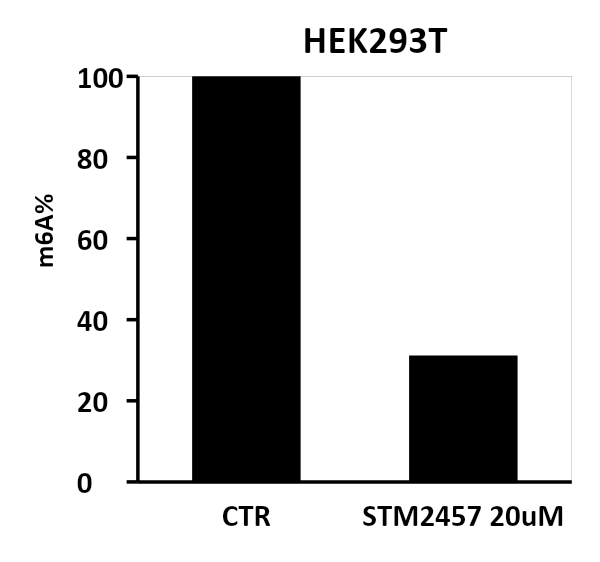


**Supplementary Figure S9 – Quantification of m6A depletion in HEK293T cells.** Quantification of m6A+ RNA levels in HEK293T cells treated with ETOH 100% and HEK293T cells treated with 20 uM STM2457 (METTL3 inhibitor from Selleckchem) for 24 h. Absorbance was measured at 450 nm.


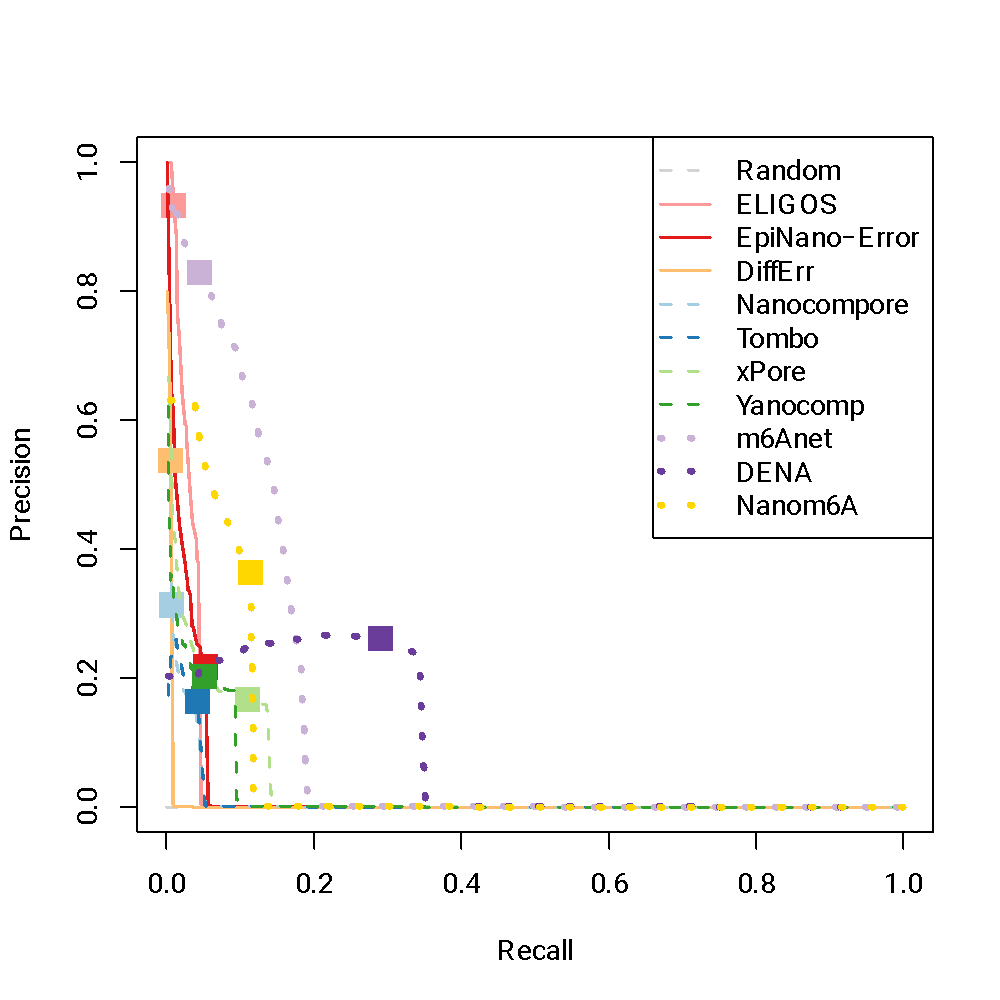


**Supplementary Figure S10 – Impact of restricting precision and recall curves to chr1.** Precision and recall curves at different cutoff values for chr1 of the mouse dataset; for each tool, the default cutoff is indicated by a square; the performance of a random classifier is included. To be compared with Figure 4G reporting the same analysis performed on the whole mouse genome.

**Supplementary Information**

**Datasets**

The *synthetic oligos dataset* is composed of four sequences that are ~2.5 kb each, with two replicates that are fully m6A-modified, totalling 660,986 reads and two replicates that are unmodified, totalling 808,283 reads [36] (**Table S2**). Compared to other datasets used in this study, this one has the advantage of having a ground truth set of m6A hits, whose position is known by design due to the synthetic origin of the oligos and the complete incorporation of m6A. At the same time, m6A hits sequence context and stoichiometry are different from biological conditions, therefore, performances observed with this dataset could be not fully representative of performances on biological datasets. This dataset was released as part of the study describing EpiNano, where the authors demonstrated that systematic errors and decreased base-calling qualities in native RNA sequencing could be used to detect m6A RNA modifications [36]. On this dataset, we were able to run the following tools: DiffErr, DRUMMER, ELIGOS, EpiNano-Error, EpiNano-SVM, MINES, m6Anet, Nanocompore, Nanom6A, Tombo, xPore and Yanocomp.

The *yeast dataset* has three replicates for both WT *S. cerevisiae* SK1 strain, totalling 7,123,187 reads, and three replicates for an IME4 KO, the only known yeast m6A writer, totalling 6,690,721 reads (**Table S2**). This dataset was obtained from [41], where it was used to test the Nanocompore m6A detection tool. The yeast dataset is characterised by a simpler and smaller transcriptome (8,223,847 nt), allowing for a high coverage. Moreover, prior knowledge about IME4 being the only m6A writer enables an effective knock-out, with minimal residual m6A levels. For this dataset, we could rely on MAZTER-seq [18] and m6A-seq [45] datasets for compiling an orthogonal reference set of m6A hits (**Table S3**). On this dataset, we were able to run the following tools: DiffErr, ELIGOS, MINES, m6Anet, Nanocompore, Nanom6A, Tombo, xPore and Yanocomp.

The *mouse dataset* includes two replicates for mESC WT, totalling 4,752,397 reads and one for the *Mettl3* KO counterpart [37], *Mettl3* being the main mouse mRNA m6A writer, totalling 1,527,198 reads [15] (**Table S2**). This dataset was released as part of a study where miCLIP2 was described, showing that in combination with machine learning it could improve m6A detection. The mouse dataset is characterised by a complex transcriptome (241,898,543 nt), with multiple isoforms for each gene (2.57 on average). For this dataset we could rely on miCLIP2 [15] and GLORI [23] datasets to compile an orthogonal reference set of m6A hits (**Table S3**). On this dataset, we were able to run the following tools: DENA, DiffErr, ELIGOS, EpiNano-Error, MINES, m6Anet, Nanocompore, Nanom6A, Tombo, xPore and Yanocomp.

The *human dataset* was generated as part of this work. We sequenced HEK293T WT cells with dRNA-seq on the PromethION Nanopore sequencer, generating 414,112 reads with a mean length of 1,470 nt mapping to chromosome chr1 (**Table S2**). For multi-sample tools requiring a baseline condition, we treated HEK293T cell line with the STM2457 *Mettl3* inhibitor, *Mettl3* being the main human mRNA m6A writer. After verifying a consistent reduction of m6A RNA levels, resulting in ~70% m6A reduction compared to the WT (**Supplementary Figure S9**), we sequenced HEK293T STM2457 treated cells with dRNA-seq on a PromethION flow-cell, generating 501,606 reads with a mean length of 1,427 nt mapping to chromosome chr1 (**Table S2**). This dataset is characterised by a complex transcriptome (437,455,218 nt), with multiple isoforms for each gene (4.03 on average). For this dataset, we restricted our analyses to chr1. This choice was motivated by the huge running time required by some of the tools for performing a genome-wide m6A detection on such a high-coverage dataset from a complex transcriptome. To confirm that chr1 is a representative subset of the whole genome, we compared the results obtained for the whole mouse transcriptome with those obtained by restricting tools’ hits and m6A reference set to those mapping to chr1. We were able to recapitulate the results obtained with the whole dataset, indicating that restricting to chr1 does not substantially impact the results (**Supplemental Figure S10**). For this dataset we could rely on GLORI [23] dataset to compile an orthogonal reference set of m6A hits (**Table S3**). On this dataset, we were able to run the following tools: DENA, DiffErr, ELIGOS, EpiNano-Error, EpiNano-SVM, MINES, m6Anet, Nanocompore, Nanom6A, Tombo, xPore and Yanocomp. EpiNano-Error ran successfully on the down-sampled datasets, but failed completing on the full dataset despite assigning 6 CPUs and 200 GB RAM.

**Tools execution**

Only nanoDoc failed to complete the analysis on all four datasets. The remaining tools that were not able to complete the analysis on one or more datasets (1 tool for oligos, 4 tools for yeast, 2 tools for mouse and 2 tools for human) did run successfully on one or more datasets. This allowed us to rule out any kind of installation issue. One possible reason for these failures may be ascribed to computational issues, either due to the high coverage (oligos and yeast dataset) or to transcriptome size and complexity (mouse and human datasets). These kinds of issues may be originating from software implementations that do not scale up well with increasing data to be processed. In any case, after two weeks of running and not producing any outputs, we stopped the tools’ execution, and reported the tools as “not able to complete”. Another possible reason for these failures is input file format issues, e. g. a special character in the sequence names which causes the program to crash. However, none of the failing tools provided any error messages for guiding us in troubleshooting.

**Supplementary Tables**

**Supplementary Table 1. Key features of the 14 tools for m6A detection on dRNA-seq data considered in this study**. *These tools could also be used using the transcriptome as reference.

| **Tool** | **Version** | **Reference space** | **Design** | **Training dataset** | **Ref.** |
| --- | --- | --- | --- | --- | --- |
| Tombo | 1.5.1 | Transcriptome | Multiple conditions | - | [48] |
| Yanocomp | 0.2 | Transcriptome | Multiple conditions | - | [54] |
| Nanocompore | 1.0.3 | Transcriptome | Multiple conditions | - | [41] |
| xPore | 2.0 | Transcriptome | Multiple conditions | - | [55] |
| ELIGOS | 2.1.0 | Genome | Multiple conditions | - | [37] |
| DiffErr | 0.2 | Genome | Multiple conditions | - | [29] |
| nanoDoc | - | Genome | Multiple conditions | - | [57] |
| DRUMMER | - | Genome* | Multiple conditions | - | [52] |
| EpiNano-Error | 1.2 | Genome* | Multiple conditions | - | [36] |
| EpiNano-SVM | 1.2 | Genome* | Single condition | Synthetic oligos | [36] |
| Nanom6A | - | Transcriptome | Single condition | Synthetic oligos | [56] |
| m6Anet | 1.1.0 | Transcriptome | Single condition | *H. sapiens* | [50] |
| MINES | - | Transcriptome | Single condition | *H. sapiens* | [51] |
| DENA | - | Transcriptome | Single condition | *A. thaliana* | [53] |

**Supplementary Table 2. Info and statistics for the considered dRNA-seq datasets.**

| **Name** | **Origin** | **Condition** | **Replicate** | **Num. reads** | **Num. bases** | **Mean reads**  **length (bp)** | **N50 reads**  **length (bp)** | **Ref.** |
| --- | --- | --- | --- | --- | --- | --- | --- | --- |
| Oligos | Synthetic | Modified | 1 | 535,068 | 577,583,521 | 1,079 | 1,590 | [36] |
|  |  |  | 2 | 125,930 | 117,472,721 | 933 | 1,314 |  |
|  |  | Unmodified | 1 | 745,236 | 943,590,142 | 1,266 | 1,943 |  |
|  |  |  | 2 | 63,047 | 60,418,566 | 958 | 1,457 |  |
| Yeast | S. cerevisiae SK1 | WT | 1 | 3,545,392 | 3,384,965,955 | 955 | 1,166 | [41] |
|  |  |  | 2 | 2,353,946 | 2,202,634,589 | 936 | 1,147 |  |
|  |  |  | 3 | 1,223,849 | 511,387,889 | 418 | 512 |  |
|  |  | IME4 KO | 1 | 3,518,935 | 3,301,675,711 | 938 | 1,167 |  |
|  |  |  | 2 | 1,600,929 | 1,607,175,012 | 1,004 | 1,249 |  |
|  |  |  | 3 | 1,570,857 | 739,664,587 | 471 | 556 |  |
| Mouse | mESC | WT | 1 | 3,162,968 | 1,667,353,900 | 527 | 558 | [37] |
|  |  |  | 2 | 1,589,429 | 739,857,456 | 465 | 487 |  |
|  |  | *Mettl3* KO | 1 | 1,527,198 | 669,736,515 | 439 | 457 |  |
| Human (chr1) | HEK293T | WT | 1 | 414,112 | 608,645,817 | 1,470 | 1,835 |  |
|  |  | *Mettl3* STM2457 inhibitor | 1 | 501,606 | 715,1587,416 | 1,427 | 1,777 |  |

**Supplementary Table 3. Reference m6A sets**. For the four datasets, the number of m6A hits obtained from orthogonal platforms is reported.

| **Dataset name** | **Platform/protocol** | **Num. m6A hits** | **Ref.** |
| --- | --- | --- | --- |
| Oligos | In-vitro synthesis | 2,576 | [36] |
| Yeast | m6A-seq | 1,290 | [45] |
|  | MAZTER-seq | 348 | [18] |
|  | m6A-seq OR MAZTER-seq | 1,605 | - |
| Mouse | miCLIP2 | 25,456 | [15] |
|  | GLORI | 96,311 | [23] |
|  | miCLIP2 OR GLORI | 103,870 | - |
| Human | GLORI | 24,576 | [23] |

**Supplementary Table 4. Tools settings**. Tools filtering parameters and default cut-offs are reported. In case multiple filtering parameters are available, the one that was varied to sample the precision- recall space and determine the precision / recall curves is highlighted in bold.

| **Tool** | **Version** | **Filtering parameters and default values** |
| --- | --- | --- |
| Tombo | 1.5.1 | p-value < 0.05 |
| Yanocomp | 0.2 | **fdr-threshold < 0.05;** min-ks = 0.2 |
| Nanocompore | 1.0.3 | **GMM_logit_pvalue < 0.01**; KS_dwell_pvalue < 1; abs(Logit_LOR) > 0.5 |
| xPore | 2.0 | FDR < 0.05 |
| ELIGOS | 2.1.0 | **adjPval < 0.0001**; pval < 1; OddR > 1.2 |
| DiffErr | 0.2 | FDR < 0.05 |
| nanoDoc | - | - |
| DRUMMER | - | **padj < 0.05;** abs(OR) > 1.5 |
| EpiNano-Error | 1.2 | Delta_sum_err > 0.1 |
| EpiNano-SVM | 1.2 | ProbM > 0.5 |
| Nanom6A | - | proba > 0.5 |
| m6Anet | 1.1.0 | **probability_modified > 0.9**; mod_ratio > 0 |
| MINES | - | - |
| DENA | - | m6A_ratio > 0.1 |
